# The *SF3B1*^*R625H*^ mutation promotes prolactinoma tumor progression through aberrant splicing of *DLG1*

**DOI:** 10.1186/s13046-022-02245-0

**Published:** 2022-01-17

**Authors:** Jing Guo, Chuzhong Li, Qiuyue Fang, Yulou Liu, Dawei Wang, Yiyuan Chen, Weiyan Xie, Yazhuo Zhang

**Affiliations:** 1 Beijing Neurosurgical Institute, Capital Medical University, Beijing, 100070 China; 2 Department of Neurosurgery, Beijing Tiantan Hospital affiliated to Capital Medical University, Beijing, 100070 China; 3 Beijing Institute for Brain Disorders Brain Tumor Center, Beijing, 100070 China; 4 China National Clinical Research Center for Neurological Diseases, Beijing, 100070 China

**Keywords:** *SF3B1* mutation, Prolactinomas, Alternative splicing, DLG1, Invasion

## Abstract

**Background:**

Recently, a hotspot mutation in prolactinoma was observed in splicing factor 3b subunit 1 (*SF3B1*^*R625H*^), but its functional effects and underlying molecular mechanisms remain largely unexplored.

**Methods:**

Using the CRISPR/Cas9 genome editing system and rat pituitary GH3 cells, we generated heterozygous *Sf3b1*^*R625H*^ mutant cells. Sanger and whole-genome sequencing were conducted to verify the introduction of this mutation. Transcriptome analysis was performed in *SF3B1*-wild-type versus mutant human prolactinoma samples and GH3 cells. RT-PCR and minigene reporter assays were conducted to verify aberrant splicing. The functional consequences of *SF3B1*^*R625H*^ were evaluated in vitro and in vivo. Critical makers of epithelial-mesenchymal transition and key components were detected using western blot, immunohistochemistry, and immunofluorescence. Suppressing proteins was achieved using siRNA.

**Results:**

Transcriptomic analysis of prolactinomas and heterozygous mutant cells revealed that the *SF3B1*^*R625H*^ allele led to different alterations in splicing properties, affecting different genes in different species. SF3B1^R625H^ promoted aberrant splicing and DLG1 suppression in both rat cells and human tumors. In addition, SF3B1^R625H^ and knocking down DLG1 promoted cell migration, invasion, and epithelial-mesenchymal transition through PI3K/Akt pathway.

**Conclusions:**

Our findings elucidate a mechanism through which mutant SF3B1 promotes tumor progression and may provide a potent molecular therapeutic target for prolactinomas with the *SF3B1*^*R625H*^ mutation.

**Supplementary Information:**

The online version contains supplementary material available at 10.1186/s13046-022-02245-0.

## Background

Pituitary adenomas constitute 10–20% of intracranial neoplasms, and prolactinomas is the most common subtype [1, 2]. In addition to hyperprolactinemia, some prolactinomas are characterized by tumor invasion, resistance to conventional therapy, and the high recurrence has no effective treatment [3]. Therefore, deciphering the underlying pathogenesis of prolactinoma and determining efficient treatment targets are of great significance to the clinical management of prolactinoma patients.

Alternative splicing is an essential step in the posttranscriptional regulation of gene expression. It is a complex process that diversifies the proteome by creating multiple proteins from the same gene [4, 5]. Mutations in splicing factor cause aberrant alternative splicing, leading to tumorigenesis [6]. Splicing factor 3b subunit 1 (SF3B1) is a core component of the U2 small nuclear ribonucleoprotein complex (U2 snRNP), which is essential for pre-mRNA splicing [7]. Recurrent somatic mutations in *SF3B1* have been reported in myelodysplastic syndrome (MDS), chronic lymphocytic leukemia (CLL), and some solid tumors including uveal melanoma, breast carcinoma, pancreas adenocarcinoma [8–13]. *SF3B1* mutations result in aberrantly spliced transcripts [14]. Approximately half of the aberrantly spliced mRNAs are subject to nonsense-mediated decay (NMD), which causes gene and protein production to be downregulated [15]. Our previous study reported a recurrent *SF3B1*^*R625H*^ mutation in prolactinoma, which was associated with poor progression-free survival and higher levels of prolactin (PRL) [16]. However, the underlying molecular mechanisms of this *SF3B1* mutation and its downstream cellular processes in prolactinoma remain unclear.

Here we generated a heterozygous *Sf3b1*^*R625H*^ mutant rat cell line using CRISPR/Cas9 genome editing technology. RNA sequencing analysis revealed that the aberrantly spliced mRNAs and affected genes differed significantly, although *SF3B1* was conserved between rat and human. Our results suggested that *SF3B1* mutations in prolactinoma stimulate PI3K/AKT signaling by downregulating Discs large 1 (DLG1), which was induced by aberrant splicing to enhance tumor invasion and migration.

## Methods

### Cell culture and generation of *Sf3b1* mutant R625H cells

The GH3 and MMQ rat pituitary cell line was purchased from the American Type Culture Collection (CCL-82.1 and CRL-10609; Manassas, VA, USA) and was cultured in Ham’s F12K medium in the presence of 2.5% fetal bovine serum (FBS) and 15% horse bovine serum (Gibco, Waltham, MA, USA). MCF7 and HEK293T cells were obtained from the National Infrastructure of Cell Line Resource (Beijing, China) and were cultured in DMEM (Gibco) with 10% FBS. PRL levels in cell culture supernatant were detected using an ELISA kit (BioVision, Milpitas, CA, USA) according to the manufacturer’s instructions.

The CRISPR/Cas9 gene editing system was used to generate the *Sf3b1* p.R625H (c.G1874A, c.A1875T) mutant GH3 cell line. A Sanger Centre CRISPR webtool (http://www.sanger.ac.uk/htgt/wge/) was used to identify two small guide RNAs (sgRNAs). Cas9/sgRNA-mediated DNA double-strand break and homologous recombination contributed to generated the specific mutation. The 5′ sgRNA sequence was 5′-GGCAAGATTCCTTCCTCA-3′ and the 3′ sgRNA sequence was 5′-GGTTAAGAGTACTGTTGTC-3′. The sgRNA pairs were cloned into a wild-type spCas9 and sgRNA expression plasmid. The donor plasmid (CL-GJ-016-puro-ΔTK) consisted of a puromycin resistance cassette flanked by two *loxP* sites and ~ 1 kb homology arms at both ends of the c. 1874 G > A, c. 1875 A > T mutant exon 14 (Wuhan Genecreate Biological Engineering Co. Ltd., Wuhan, China) (Fig. S1A). All the constructs were confirmed by sequencing. The Neon Transfection System (Thermo Fisher Scientific, Waltham, MA, USA) with pulse voltage of 1000 V for 40 ms was applied to co-transfect GH3 cells on a 10-cm plate with 1 μg of CL-GJ-016-puro-TK and 3 μg of Cas9/sgRNA. Puromycin (0.5 μg/mL) was added at 48-h post-transfection. Single-cell cloning was picked and cultured in a 96-well plate. Sanger sequencing was performed using genomic DNA to confirm the mutations (DIA-UP Biotech, Beijing, China). Primers were designed to amplify exon 14 of the *Sf3b1* gene (Table S1).

### Adenoviral constructs and primary culture of human prolactinomas cells

The adenoviral constructs for mutant *SF3B1*^*R625H*^ and wild-type *SFB31* were generated by BAC Biological Technology (Beijing, China). Human prolactinoma cells were prepared and cultured as described previously [16]. Tumor cells were infected with adenovirus at a multiplicity of infection of 100, and then harvested for reverse transcriptase-PCR (RT-PCR) 48-h later.

### RNA sequencing

Total RNA in GH3 cells was extracted using the AllPrep® DNA/RNA Mini kit (QIAGEN, Hilden, Germany). Sequencing libraries were generated using NEBNext® Ultra™ Directional RNA Library Prep Kit for Illumina® (NEB, Ipswich, MA, USA). The libraries were sequenced using an Illumina platform, and 150-bp paired-end reads were generated. Clean reads were mapped to the Rat Rnor_6.0 genome using Hisat2 (v2.0.5), fragments per kilobase per million mapped fragments (FPKM), and transcripts per million (TPM) for each sequenced gene.

### Differential alternative splicing and differential gene expression analyses

Differentially expressed genes (DEGs) were identified using DESeq2 using *P* < 0.05 and absolute value of fold change ≥1.5. For alterative splicing analysis, rMATS (version 4.0.2) [17] based python algorithm was used to identify alternative splicing events by quantifying exon-exon junction spanning reads on annotated splice junctions in rat GENCODE Rnor_6.0 assembly. Differentially spliced mRNAs were defined as FDR < 0.05 and a minimum inclusion level difference > 10% or < − 10%. Three mutant GH3 replicates and three wild-type replicates were compared. Data of two prolactinoma patients with the *SF3B1*^*R625H*^ mutation and two wild-type cases were selected for alternative splicing analysis, and two mutant and 13 wild-type cases from our previously reports were selected for differential expression analysis (Table S2) [16]. Functional enrichment analysis was performed using Gene Ontology (GO) and the Kyoto Encyclopedia of Genes and Genomes (KEGG) via R package clusterProfiler to predict the biological functions of DEGs and differentially spliced transcripts. The GO terms analyzed included biological process, cellular components, and molecular functions. A KEGG pathway with a *P* value < 0.05 was considered as statistical significance.

### Whole-genome sequencing and variant identification

Genomic DNA was extracted from wild-type and mutant GH3 cells using the Blood & Cell Culture DNA Mini Kit (QIAGEN). DNA was sequenced using an Illumina HiSeq 2000, generating 150-bp paired reads. Reads were aligned to the Rat Rnor_6.0 genome using Burrows–Wheeler Aligner (BWA) [18]. SAMtools was used to generate BAM files [19]. Visual inspection of the *Sf3b1* R625H mutation was performed using the Integrative Genomics Viewer [20].

### Minigene assay

A DNA fragment containing the *DLG1* exon 22 genomic sequence with 151 bp flanking intron 21 and 271 bp flanking intron 22 was inserted between the *KpnI* and *EcoRI* restriction sites of the pcMINI vector to produce the DLG1 minigene construct. Sanger sequencing was performed to confirm the sequence of the inserted fragment. Briefly, the *DLG1* minigene and an adenoviral vector were co-transfected into 293 T cells. After 48 h, RNA was harvested. PCR products were separated by 2% agarose gels and were confirmed by Sanger sequencing. The primers used for the spliced products were: 5′-CTAGAGAACCCACTGCTTAC-3′ (forward) and 5′-TAGAAGGCACAGTCGAGG-3′ (reverse).

### Transfection

RIBOBIO (Guangdong, China) synthesized the small interfering (si) RNA duplexes; siRNA sequences of rat and human *DLG1* are listed in Table S3. The *Dlg1* overexpressed plasmid, pLV-hef1a-Puro-WPRE-CMV-Dlg1–3 × FLAG, was constructed by Beijing Syngentech Co., Ltd. (Beijing, China). All transfections (siRNAs and overexpression plasmids) were performed using Lipofectamine® 3000 Transfection Reagent (Invitrogen, Carlsbad, CA, USA) according to the manufacturer’s protocols. Cells were transfected with siRNA or plasmids for 48–72 h and were harvested to perform quantitative (q) PCR and western blot.

### RT-PCR and qPCR

The RNeasy Mini Kit (QIAGEN) was used to extract total RNA and the High Capacity cDNA Reverse Transcription Kit (Thermo Fisher Scientific) was used to generate cDNA, both according to the manufacturers’ instructions. RT-PCR was performed using I-5 High-Fidelity Master Mix (MCLAB, San Francisco, CA, USA). PCR products were separated by 1–3% agarose gels. All qPCR assays were performed using Power SYBR™ Green PCR Master Mix (Thermo Fisher Scientific) and were analyzed using QuantStudio 3 and 5 systems (Applied Biosystems, Waltham, MA, USA). The comparative Ct method was used to evaluate relative gene expression. Primers were listed in Table S1.

### Scanning electron microscopy (SEM)

Cells were collected and fixed with 2.5% glutaraldehyde (Solarbio, Beijing, China) at 4 °C overnight for SEM preparation. After rinsing with PBS and sterile water, the cells were dehydrated using an ethanol gradient. The samples were coated with gold after critical point drying. Pictures were obtained using a Hitachi SU8020 SEM (Tokyo, Japan).

### Cell migration and wound-healing assays

Cell migration culture dish inserts from Ibidi (Martinsried, Germany) were used for wound-healing assays. After 24 h of transfection, cells were seeded into the chambers of the culture dish inserts. The inserts were removed on the next day, and fresh culture medium was added to each well. Scratches were photographed at different points in time using a Zeiss microscope (Oberkochen, Germany).

*Sf3b1* mutant and wild-type GH3 cells were seeded onto Imagelock 96-well plates (Essen Bioscience, Ann Arbor, MI, USA). The IncuCyte® Wound Maker (Essen Bioscience) was used to make uniform wounds in a monolayer of confluent cells. Phase contrast imaging was performed every 12 h till 96 h. Images were analyzed using IncuCyte® S3 2018B-2019A software (Essen Bioscience), and data were analyzed using GraphPad Prism7 (GraphPad Software, Inc., La Jolla, CA, USA).

### Transwell assays

Transwell plates and Matrigel-coated transwell plates (Corning-Costar, Corning, NY, USA) were used to determine cell migration and invasion capabilities, respectively. Briefly, HEK293T cells resuspended in serum-free medium were inoculate into the upper chamber, and DMEM with 10% FBS was placed in the bottom chamber. After 24 h, cells on the bottom surface of the chamber were stained with crystal violet and counted under a microscope (Zeiss).

### Western blot analysis

Protein samples were separated on 8–10% Bis-Tris SDS-PAGE gels and transferred to polyvinylidene fluoride membranes (Merk, Kenilworth, NJ, USA). All primary antibodies (Table S4) were diluted in TBST containing 1% bovine serum albumin (BSA) and were incubated with the membranes overnight at 4 °C. Immunoreactive bands were visualized using chemiluminescence.

### Immunohistochemistry

Human prolactinoma tumor specimens were used to examine DLG1, E-cadherin, N-cadherin, and Snail protein levels. The primary antibodies were summarized in Table S4. Immunohistochemistry was performed by the Leica Bond Polymer Refine Detection system (Leica Biosystems, Wetzlar, Germany). All slides were scanned into digital images, and expression was examined using Aperio AT2 (Leica Biosystems). Staining intensity was scored as 0 (negative), 1 (weak), 2 (moderate), or 3 (strong). The percentage of immunostaining was recorded, and H-scores were calculated using the formula: H-score = 1 × (% weakly stained cells) + 2 × (% moderately stained cells) + 3 × (% strongly stained cells)], ranging from 0 to 300.

### Rat prolactinoma model

Rat pituitary tumors were induced by subcutaneously implanting 1-cm silastic capsules containing 10 mg of 17-β estradiol in 4-week-old female F344 rats. Prolactinomas were induced by 17β-estradiol for 5 weeks, as described previously [21]. All experimental protocols were approved by the Animal Use and Care Committee of Beijing Tiantan Hospital. Prolactinomas were validated via 7.0-T magnetic resonance imaging (MRI) before intra-pituitary injection. The rats were anaesthetized and adenovirus vector control, wild-type *SFB31*, or *SF3B1*^*R625H*^ (1 μl) was stereotactically injected into each bilateral tumor. Another MRI was performed 2 weeks later.

### Immunofluorescence staining of rat tumor tissues

Rats were anaesthetized and heart-perfused with 4% formalin. Tumor tissues were collected and immersed in 10% sucrose for 2 h, followed by 30% sucrose incubation overnight before freezing in OCT compound. Frozen 5-μm-thick sections were fixed in ice-cold acetone. Slides were blocked with goat serum and incubated with primary antibodies overnight at 4 °C (summarized in Table S4). After washing with PBS, the slides were incubated in Alexa Fluor 488 and 594 secondary antibodies (Invitrogen) for 1 h at room temperature; DAPI was used to visualize nuclei.

### Phalloidin staining and confocal microscopy

Cells were plated on confocal dishes coated with poly-L-lysine. After incubation at 37 °C for 24 h, cells were fixed with 4% paraformaldehyde for 30 min, and were stained with 5 μg/mL Alexa Fluor 488-phalloidin (Invitrogen) to reveal filamentous actin (F-actin) in PBS for 40 min at 37 °C. DAPI was used to visualize nuclei, and images were captured by confocal laser-scanning microscopy (Zeiss).

### Statistical analysis

All statistical analyses were performed using R v3.4.1 (https://www.r-project.org/) and Prism 7 (GraphPad Software, Inc.). All experiments were performed with at least three biological replicates, and all quantitative data represent mean ± standard deviation (SD). Statistical significance was determined by unpaired Student’s *t* test (two groups) or one-way ANOVA (multiple groups). *P* < 0.05 was considered statistically significant.

## Results

### Generation of *Sf3b1* R625H-mutant cells

**Fig. 1:**
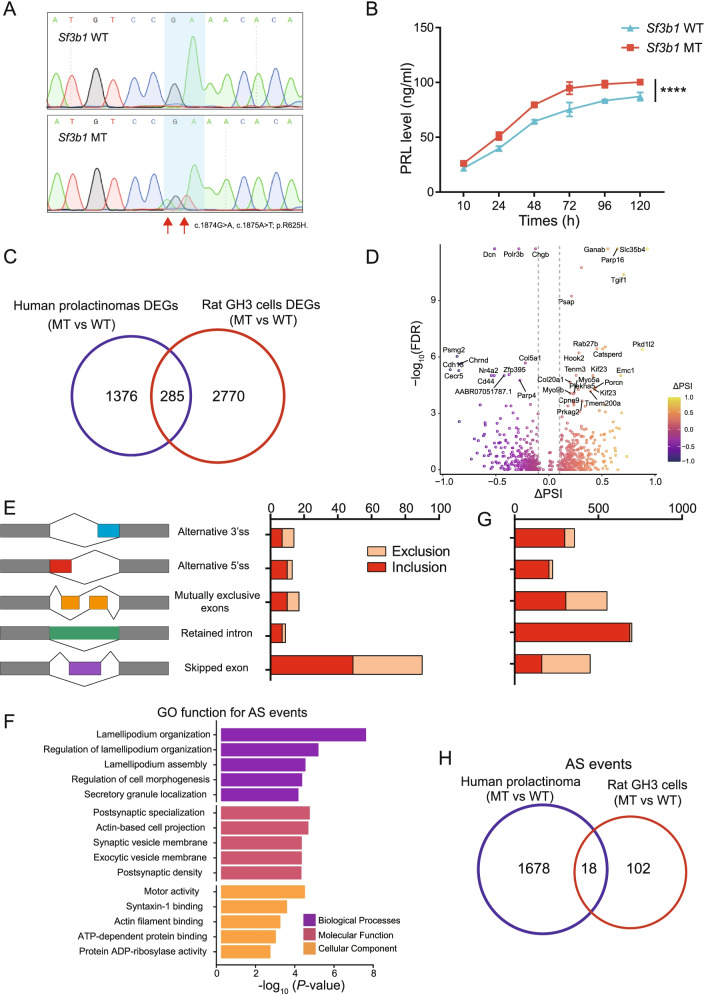
Generation of heterozygous *Sf3b1*-R625H mutant GH3 cells. **A** Sequencing chromatogram demonstrating the heterozygous *Sf3b1*-R625H mutation. The homozygous nucleotide in *Sf3b1* wild-type GH3 cells and heterozygous nucleotides in *Sf3b1* mutant GH3 cells are shown. **B** PRL secretion in *Sf3b1* wild-type and mutant GH3 cells are shown. Data are represented as mean ± SD. (*P*-values by two-way ANOVA, ****P < 0.0001). **C** Venn diagram showing the overlapping DEGs identified in *SF3B1* mutant human prolactinoma and GH3 cells. **D** Volcano plot showing the differential alternative splicing events in *Sf3b1* mutant vs. wild-type GH3 cells. Significant alternative splicing events identified at FDR < 0.05 and |∆PSI| > 0.1. PSI: Percent Splice In. Top 30 significant mis-spliced genes are indicated. **E** Number and type of mis-splicing events in *Sf3b1* mutant vs. wild-type GH3 cells are shown. Inclusion: events with ΔPSI> 10%; exclusion: events with ΔPSI<− 10% **F** GO analysis of mis-spliced genes in *Sf3b1* mutant vs. wild-type GH3 cells; the top five ranked terms are shown. **G** Number and type of mis-splicing events in *SF3B1* mutant vs. wild-type human prolactinoma. **H** Venn diagram showing the overlap of mis-spliced genes identified in *SF3B1* mutant human prolactinoma and GH3 cells

Our previous study identified the somatic hotspot mutation *SF3B1*^*R625H*^ occurred in 19.8% of prolactinomas [16]. To investigate the biological role of the *SF3B1*^*R625H*^ mutation in prolactinoma progression as well as similarities and differences between human and rats, we introduced the *Sf3b1*^*R625H*^ missense mutation into the rat pituitary cell line GH3 using the CRISPR/Cas9 gene editing system (Fig. S1A). After isolating and expanding single cell clones, a heterozygous *Sf3b1*-R625H mutation in GH3 cells was confirmed by Sanger sequencing (Fig. 1A). Whole-exome sequencing analyses of the mutant cell line also validated the heterozygous *Sf3b1* mutation, p.R625H (NC_005108.4:g.61608130 T > A, g.61608131C > T, NM_053426.1:c.1874G > A, c.1875A > T), and the frequency of the mutant allele obtained from read counts was 28.57% (18 A&T and 45 G&A, Fig. S1B). Additionally, to determine whether heterozygous *Sf3b1*-R625H mutation status affected PRL secretions, we performed ELISA to test PRL levels in culture media from wild-type and mutant cells. Consistent with our previous results in mutant human prolactinoma [16], PRL levels in supernatant from *Sf3b1* mutant cells was elevated compared to wild-type (Fig. 1B). Together, these data suggested successful construction of a rat cell line model harboring the *Sf3b1*-R625H mutation was able to exhibit *SF3B1*-mutant prolactinoma characteristics.

### Heterozygous *Sf3b1*-R625H status altered the transcriptome of GH3 cells

To explore transcriptome-level alterations in *Sf3b1*-R625H mutant cells, we conducted RNA-seq analysis from wild-type and mutant cultured cells. Using *P* < 0.05 and |fold change| > 1.5, we identified 3058 DEGs: 1271 upregulated and 1787 downregulated genes (Fig. S2A; Table S5). Clustering analysis showed distinctly different gene expression patterns between wild-type and *Sf3b1*-mutant cells (Fig. S2B). Moreover, compared with *SF3B1*-wild-type patient samples (*n* = 13), we identified 1062 downregulated and 599 upregulated genes in *SF3B1*-mutant human prolactinomas (*n* = 2) with *P* < 0.05 and |fold change| > 1.5 (Fig. S2C-D; Table S6). We found that only approximately 9% (285/3055) of the DEGs overlapped between rat GH3 cells and human prolactinoma samples (Fig. 1C).

Previous studies have described that the *SF3B1* mutations caused alternative splicing defects results in incorrect recognition of the 3′ splice site, generating aberrant transcripts [15, 22, 23]. To determine whether *Sf3b1*-R625H is associated with mutation-specific alterations in pre-mRNA splicing, we performed percent spliced-in (PSI) analyses of alternative splicing using the rMATS tool [17]. At a False Discovery Rate (FDR) of < 0.05 and |ΔPSI| > 0.1, we obtained 143 alternative splicing events in *Sf3b1*-R625H cells (Fig. 1D, Table S7), with exon skipping were the most frequent event (Fig. 1E). GO functional analysis of the alternatively spliced genes revealed enrichment of ontologies including lamellipodium organization, regulation of cell morphogenesis, and motor activity (Fig. 1F, Table S8). In addition, we also performed end point RT-PCR to confirm several alternative splicing-associated changes (Fig. S3A-N). Re-analysis of RNA-seq data generated from two *SF3B1*-mutant and two *SF3B1*-wild-type prolactinoma cases [16] identified 2287 alternative splicing events in *SF3B1*-mutant cases, with a higher proportion of inclusion alternative 3′ splice site (ΔPSI> 10%) and inclusion retained intron (ΔPSI> 10%) events (Fig. 1G; Fig. S4A-B; Table S9–10). Consistent with human data, *Sf3b1* mutant GH3 cells had more inclusion (*n* = 83, ΔPSI> 10%) than exclusion (*n* = 60, ΔPSI<− 10%) alternative splicing events compared with wild-type cells (Fig. 1E and G). Furthermore, only approximately 15% (18/120) of the aberrantly spliced genes in rat *Sf3b1*-mutant GH3 cells were found in human samples (Fig. 1H). These data indicated that the limited conservation between rat and human intronic sequences caused the mutant SF3B1 to have different effects on alternative mRNA splicing in the two species.

### The R625H mutation induced epithelial-mesenchymal transition (EMT) phenotypes in tumor cells

**Fig. 2:**
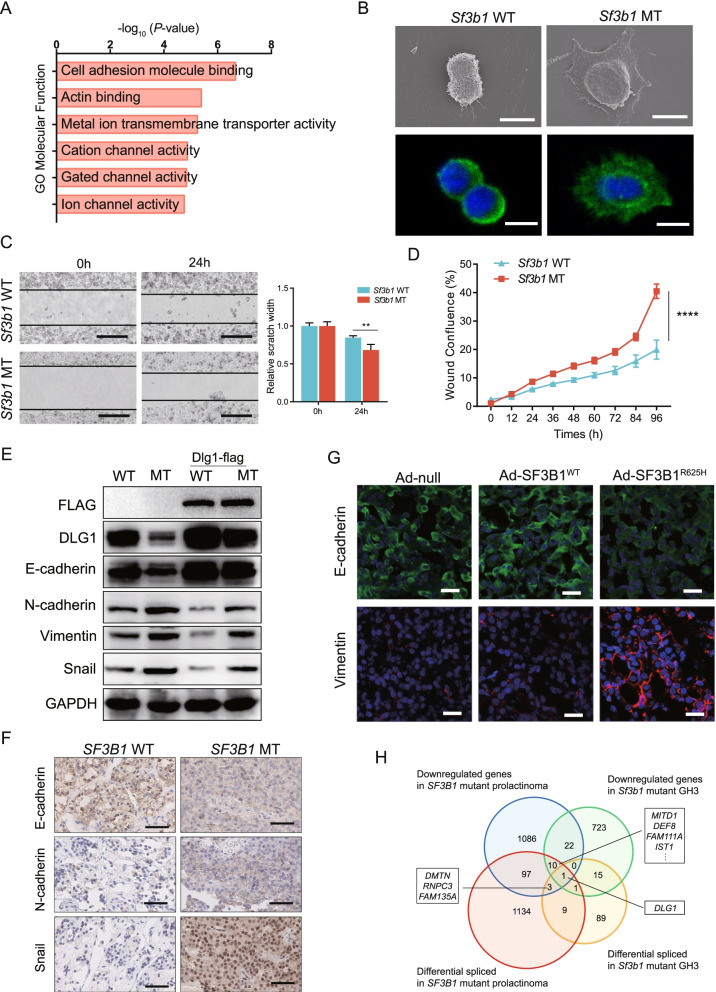
Mutant SF3B1 promoted EMT phenotypes in prolactinoma. **A** GO functional enrichment analysis of DEGs in *Sf3b1* mutant vs. wild-type GH3 cells. **B** Scanning electron microscopy (SEM; upper) and confocal images of the actin cytoskeleton stained with rhodamine–phalloidin (lower) of *Sf3b1* wild-type and mutant GH3 cells; scale bar: 10 μm. **C** Scratch wound healing assays were performed using *Sf3b1* wild-type and mutant GH3 cells; scale bar: 200 μm. Relative scratch widths are shown over time (*n* = 3; mean ± SD shown below; *P* values by Student’s test). **D** Kinetics of wound confluence over 96 h were analyzed using IncuCyte 2018B software (Essen Bioscience). The results are expressed as mean ± SD, *n* = 3. *P* values by two-way ANOVA; ****P < 0.0001. **E** DLG1, E-cadherin, N-cadherin, Vimentin, and Snail levels in *Sf3b1* wild-type and mutant GH3 cells transduced with or without Dlg1-FLAG cDNA. **G** Immunofluorescence staining of E-cadherin and Vimentin in rat prolactinoma tumors stereotactically injected with Ad-null, Ad-SF3B1^WT^, and Ad-SF3B1^R625H^; scale bar: 50 μm. **F** Representative immunohistochemical staining for E-cadherin, N-cadherin, and Snail in *SF3B1* mutant and wild-type human prolactinoma tumors; scale bar: 50 μm. **H** Venn diagram of the numbers of differentially spliced (q < 0.05; t test) and differentially expressed genes (q < 0.05; DESeq2) in *SF3B1* mutant human prolactinoma and GH3 samples

Our previous study found that most *SF3B1*-mutant tumors exhibit invasive behavior, which is associated with poor progression-free survival in prolactinoma [24]. Furthermore, GO enrichment analysis of the DEGs and alternative splicing events revealed that cell adhesion molecules and lamellipodium organization were significantly altered in *Sf3b1*-mutant GH3 cells (Figs. 2A and 1F, Tables S8 and S11). These results indicated that *SFB31*-mutant tumor cells exhibit invasive behaviors. To examine changes in the adhesive properties of mutant cells, SEM was performed. Compared with round *Sf3b1* wild-type cells, *Sf3b1* mutant cells had prominent lamellipodial extensions (Fig. 2B). Moreover, F-actin polymerization and filopodium formation were observed in *Sf3b1* mutant cells (Fig. 2B). Lamellipodia formation and dramatic reorganization of the actin cytoskeleton are involved in EMT, which is associated with tumor cell invasion and metastasis [25, 26]. To explore whether *Sf3b1* mutation affects cell migration, wound-healing assays were conducted. Migration ability of *Sf3b1* mutant cells was significantly enhanced compared to the wild-type cells (Fig. 2C). These effects on migration was also confirmed by the IncuCyte ZOOM® 96-well Scratch Wound cell migration assay (Fig. 2D).

To determine whether the *Sf3b1* mutation modulated cell migration and invasion through the EMT pathway, we examined EMT makers. We observed a significant reduction in epithelial maker and an increase in mesenchymal maker expressions in mutant cells. E-cadherin were downregulated, but N-cadherin, Vimentin, and Snail were elevated in mutant cells (Fig. 2E). Consistently, immunohistochemistry of sections from wild-type and mutant prolactinoma tissues supported these findings (Fig. 2F). E-cadherin expression levels yielded an H-score of 146.7 ± 17.64 in wild-type samples and 53.3 ± 14.53 in mutant samples (*P* = 0.015). The H-scores of Snail were 20 ± 5.78 and 196.7 ± 20.28, respectively (*P* = 0.0011). The H-scores of N-cadherin were 15 ± 2.89 and 76.67 ± 8.82, respectively (*P* = 0.0027). To explore whether *SF3B1*-R625H promoted prolactinoma EMT in vivo, F344 rat prolactinomas were induced by 5-week 17β-estradiol treatment. Then the rat prolactinomas were stereotactically injected with wild-type and R625H *SF3B1* encoding adenoviruses and negative controls (Fig. S4C). After 2 weeks, tumors were collected and immunofluorescence was used to examine EMT maker expression. Decreased levels of E-cadherin and increased levels of Vimentin were observed in the R625H groups (Fig. 2G). Overall, these data suggested that mutant SF3B1 promoted EMT.

Next, to explore the underlying molecular mechanisms of *SF3B1* mutation induced EMT, we analyzed the intersection of aberrantly spliced genes and DEGs in rat GH3 cells and human prolactinoma samples (Fig. 2H). From this intersection, *DLG1* was the only identified gene (Fig. 2H), and was significantly downregulated and mis-spliced in *SF3B1* mutants (Tables S5, S6, S7 and S9).

### Mutant SF3B1 caused *DLG1* aberrant splicing

**Fig. 3:**
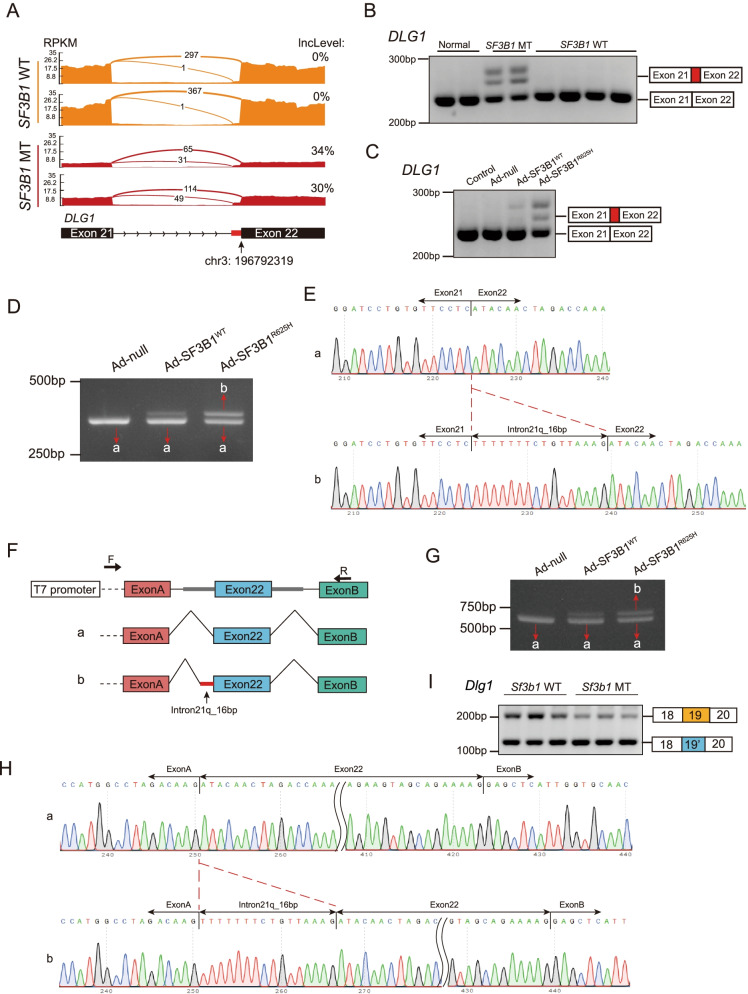
Mutant SF3B1 caused aberrant splicing of *DLG1*. **A** Sashimi plots of alternative 3′ splice sites of *DLG1* in representative human prolactinoma with or without *SF3B1* mutations. **B** RT-PCR of *DLG1* splicing events in human prolactinoma samples with or without *SF3B1* mutation. The 3′ splice sites used are shown in the schematic. **C** RT-PCR results of aberrant *DLG1* splicing in primary human prolactinoma cells infected with adenovirus carrying *SF3B1*-WT and *SF3B1*-R625H vectors. **D** RT-PCR of *DLG1* splicing in 293 T cells infected with adenovirus carrying *SF3B1*-WT and *SF3B1*-R625H vectors. Band a: canonical *DLG1* transcript; Band b: aberrant *DLG1* transcript. **E** The sequences of gel-purified fragments showing the canonical *DLG1* transcript (a) and aberrant transcript (b). **F** Schematic diagram of the minigene construct and schematic diagram of Sanger sequencing of RT-PCR products. **G** RT-PCR results of *DLG1* splicing in 293 T cells co-transduced with the minigene reporter and adenovirus carrying *SF3B1*-WT and *SF3B1*-R625H vectors. **H** The sequence of gel-purified fragments. The canonical *DLG1* transcript (a) and aberrant *DLG1* transcript (b) were observed in the *SF3B1*-WT and *SF3B1*-R625 groups, respectively. **I** RT-PCR results of *Dlg1* splicing in *Sf3b1* mutant GH3 cells. The mutually exclusive splicing type is shown in the schematic

DLG1 is a member of the molecular scaffold protein family known as membrane associated guanylate kinases (MAGUKs) [27]. DLG1 is a tumor suppressor which is associated with the establishment and maintenance of cell polarity [28, 29]. To investigate whether *SF3B1* mutation could cause *DLG1* aberrant splicing, RNA-seq data from human prolactinoma and GH3 cells was examined. Across human prolactinoma, aberrant splicing of *DLG1* was induced by mutant SF3B1 through usage of a cryptic 3′ splice site (Fig. 3A; Table S9), which was confirmed by end point RT-PCR in prolactinoma patient samples (Fig. 3B). To verify the effect of mutant SF3B1 on *DLG1* in vitro, we infected primary cultured pituitary tumor cells with adenovirus carrying the *SF3B1*-R625H mutation, and the results showed that the cryptic *DLG1* transcript was observed in the Ad-SF3B1-R625H group (Fig. 3C). Similar results were observed in HEK293T cells (Fig. 3D and S5A). To examine differences between the wild-type and mutant, we performed thymine-adenine (TA) cloning. RT-PCR and sequencing clones confirmed that the Ad-SF3B1-R625H group contained more abnormal PCR products than the Ad-SF3B1-WT group (Fig. S5B). The expected fragment with a 16-bp extension of exon 22 was identified using Sanger sequencing of PCR products (Fig. 3E). Next, we generated a minigene construct that contained sequences from the misspliced intron and flanking exon to explore alternative splicing (Fig. 3F). A complete spliced RNA containing exon 22 was observed in the *SF3B1*-WT and control groups (Fig. 3G, left and middle lanes). In contrast, a larger transcript was found in the *SF3B1*-R625H group that retained an extra 16 bp (Fig. 3G, right lane). The extra 16-bp fragment was confirmed by Sanger sequencing (Fig. 3H). Additionally, mutually exclusive splicing of *Dlg1* in *Sf3b1*-mutant GH3 cells was confirmed by RT-PCR (Fig. 3I and S5C; Table S7). These data indicated that mutant SF3B1 induced *DLG1* aberrant splicing.

**Fig. 4:**
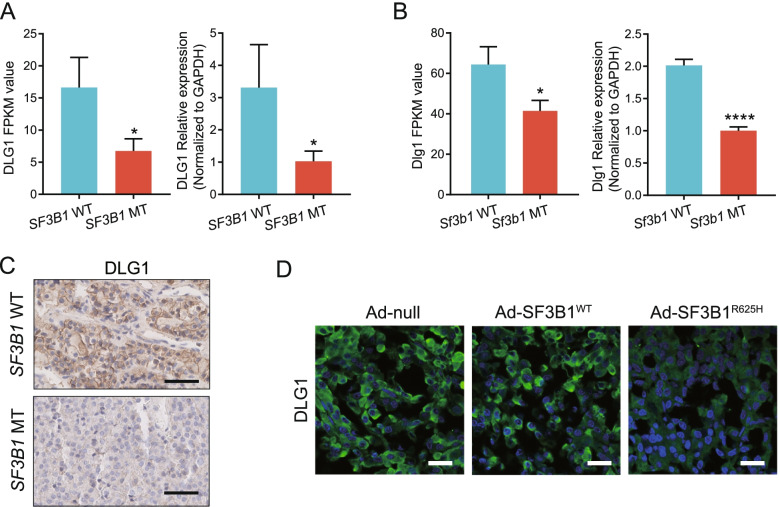
Mutant SF3B1 altered DLG1 expression. **A***DLG1* mRNA expression (RNA-seq data and quantitative PCR) in *SF3B1* mutant (*n* = 2) vs. wild-type (*n* = 13) human prolactinoma tumors. **B***DLG1* mRNA expression in *Sf3b1* mutant (*n* = 3) versus wild-type (*n* = 3) GH3 cells. FPKM: Fragments per kilobase per million mapped fragments. The results are expressed as mean ± SD (Student’s t test, *P < 0.05, ****P < 0.0001). **C** Representative immunohistochemical staining for DLG1 in *SF3B1* mutant and wild-type human prolactinoma tumors; scale bar: 50 μm. **D** Immunofluorescence staining for DLG1 in rat prolactinoma tumors stereotactically injected with Ad-null, Ad-SF3B1^WT^, and Ad-SF3B1^R625H^, respectively; scale bar: 50 μm

To verify whether the *SF3B1* mutation altered *DLG1* expression, RNA-seq data was examined and qPCR was performed in *SF3B1* mutant samples. Our results revealed that *DLG1* was downregulated in *SF3B1*-mutant human prolactinoma samples and GH3 cells (Fig. 4A-B). Furthermore, immunohistochemistry and western blotting revealed that DLG1 protein expression was decreased in *SF3B1*-mutant human samples, GH3 cells and MMQ cells (Fig. 4C and 2E, Fig. S5D). The H-scores of DLG1 were 153 ± 17.64 in *SF3B1*-wild-type and 40 ± 20.82 in *SF3B1*-mutant human tissues (*P* = 0.0142). To further confirm the effects of the *SF3B1* mutation on DLG1 expression, immunofluorescence was performed on tumors stereotactically injected with adenoviruses. Decreased DLG1 expression was observed in the Ad-SF3B1 R625H group (Fig. 4D). Taken together, these data suggested that mutant SF3B1 induced *DLG1* aberrant splicing and reduced DLG1 expression.

### Knocking down *DLG1* promoted cell migration, invasion, and EMT

**Fig. 5:**
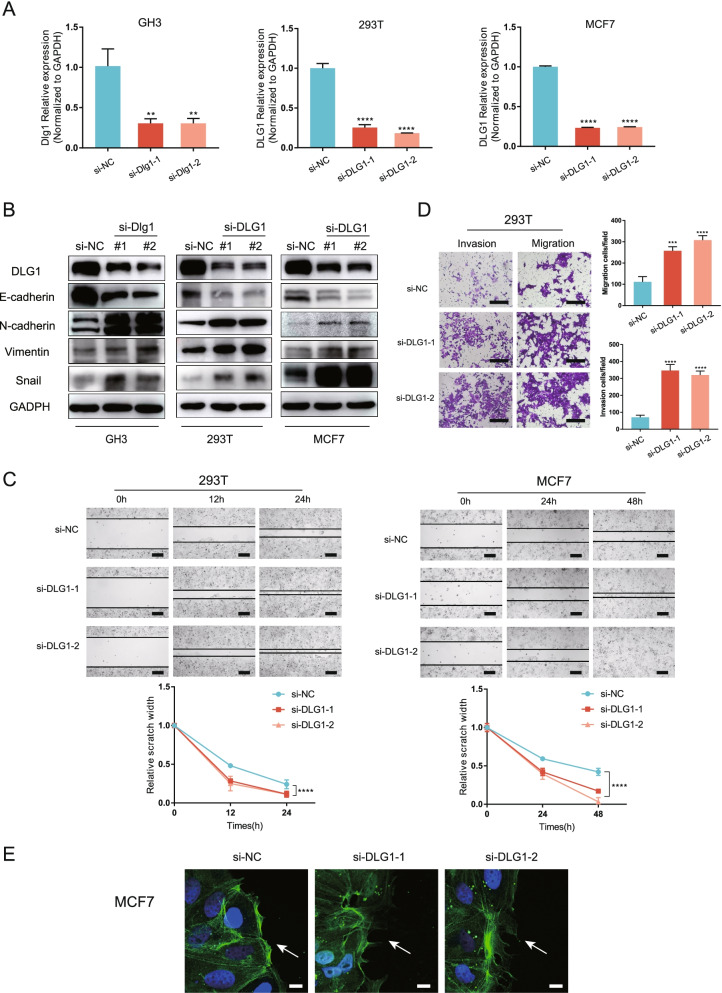
Knocking down DLG1 promoted cell migration, invasion, and EMT. **A** Quantitative PCR analysis of DLG1 knockdown efficiency in GH3, MCF-7, and 293 T cells. Results are expressed as mean ± SD; *P* values by one-way ANOVA compared with control (si-NC). **B** DLG1 knockdown significantly increased N-cadherin, Vimentin, and Snail expression but decreased E-cadherin expression in GH3, MCF-7, and 293 T cells. **C** Scratch assay of 293 T and MCF7 cells with or without DLG1 siRNA; scale bar: 200 μm. Relative scratch width over time shown below (*n* = 3; mean ± SD shown below; *P* values by two-way ANOVA). **D** Transwell assay of 293 T cells transfected with negative control (si-NC) and si-DLG1; scale bar: 200 μm. Data are presented as the mean ± SD from three independent experiments. *P* values by one-way ANOVA; **P < 0.01; ***P < 0.001; ****P < 0.0001, relative to control (si-NC). **E** Confocal immunofluorescence images in wound healing migration assays of MCF7 cells. After transfection with siRNAs for 48 h, MCF7 cells were scratched to allow cell migration. Cells were fixed after migrating for 12 h, and then stained with phalloidin (green) and DAPI (blue) to reveal F actin and nuclei, respectively; scale bar: 50 μm

To evaluate the roles of DLG1 in invasion and migration, we knocked down *DLG1* using siRNAs. Both the mRNA and protein levels of DLG1 were efficiently depleted in GH3, 293 T, and MCF7 cells (Fig. 5A-B). Suppressing DLG1 decreased the expression of E-cadherin and increased the expression of N-cadherin, Vimentin, and Snail (Fig. 5B), suggesting that EMT was enhanced. Furthermore, restoring DLG1 expression in wild-type and mutant GH3 cells inhibited EMT processes (Fig. 2E). Wound healing assays revealed that si-DLG1-transfected cells traversed wounds significantly faster than the control group (Fig. 5C). Comparable migration differences were also observed in the transwell assay (Fig. 5D). In transwell Matrigel invasion assays, DLG1 knockdown promoted cells migration through the Matrigel layer (Fig. 5D). Furthermore, phalloidin staining showed differences in F-actin organization at the wound margin. Compared with si-NC-transfected cells, si-DLG1-transfected cells showed polarized lamellipodia formation via F-actin (phalloidin) staining (Fig. 5E). These results suggested that depleting DLG1 enhanced tumor cell migration and invasion, and promoted EMT.

### The SFB31/DLG1 axis promoted tumor invasion via the PI3K/AKT pathway

**Fig. 6:**
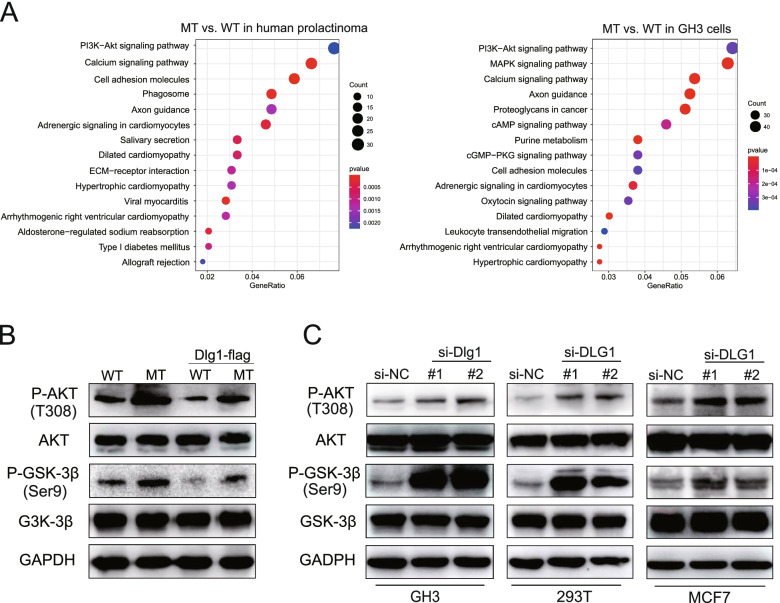
The SFB31/DLG1 axis promoted tumor invasion via the PI3K/AKT signaling pathway. **A** KEGG enrichment analysis of DEGs in *SF3B1* mutant vs. wild-type human prolactinoma and GH3 cells. **B** Phosphorylated and total AKT and GSK-3β levels in *Sf3b1* WT and mutant GH3 cells transduced with or without Dlg1-FLAG cDNA. **C** Phosphorylated and total AKT and GSK-3β levels in GH3, 293 T, and MCF7 cells transduced with or without DLG1 siRNA

To determine the key biological mechanism through which mutant SF3B1 promoted prolactinoma progression, we performed a function enrichment analysis of the DEGs from human tumors and rat GH3 cells. We found that PI3K/AKT signaling was significantly altered in mutant SF3B1 samples compared with wild type samples (Fig. 6A, Tables S12–13). We therefore detected the relevant effectors within this pathway through western blotting and found increased phosphorylation of AKT (T308) and GSK-3β in *Sf3b1*-mutant GH3 cells (Fig. 6B). To clarify the specific role of DLG1 in PI3K/AKT pathway, we manipulated DLG1 levels in GH3, 293 T, and MCF7 cells. Knocking down *DLG1* increased phosphorylation of AKT and GSK-3β (Fig. 6C). Furthermore, restoration of DLG1 expression in wild-type and mutant GH3 cells showed reduced phosphorylation of AKT and GSK-3β (Fig. 6B). Together, these data suggested that mutant SF3B1 alters DLG1 levels and promotes PI3K/AKT signaling in prolactinoma.

## Discussion

*SF3B1* is the most frequently mutated splicing gene in cancer, and its three major hotspots are: K700, R625, and R622 [30]. Among these mutated codons, K700E is the most common mutation in CLL, MDS, acute myeloid leukemia, and invasive breast carcinoma, while R625 is the predominant mutation in uveal melanoma and skin cutaneous melanoma [31]. Previous studies have explored how the *SF3B1*^*K700E*^ mutation contributes to aberrant splicing and downstream pro-tumorigenesis mechanisms in CLL and breast cancer using cell lines and mouse disease models [31, 32]. To understand the molecular and phenotypic consequences of the *SF3B1*^*R625H*^ mutation on prolactinoma tumorigenesis, we generated a heterozygous *Sf3b1*-R625H mutant rat pituitary cell line. Our results indicated that heterozygosity for *Sf3b1*-R625H in GH3 cells was able to exhibit human prolactinoma characteristics, including higher PRL levels and invasive behaviors. Although phylogenetic analysis revealed that the *SF3B1*^*R625H*^ locus is highly conserved across species [16], our results found that only a few splicing events were shared between mutant human prolactinoma and rat cells. These results may be caused by the frequency of the mutant allele in heterozygous mutant cells and limited conservation of intronic sequences between rats and humans. Moreover, our study found that most aberrant splicing events in *SF3B1* mutant human prolactinoma were retained intron and the alternative 3′ splice site. This is different from previous observations in *SF3B1*-mutant cancers [15, 33], which suggested a higher proportion of alternative 3′ splice site events, but were similar to a recent report in *SF3B1*-mutant MDS [14]. Further studies with larger samples are necessary to validate the splicing events. In particular, we found that similar downregulation and missplicing of *DLG1* transcript in both rat cells and human prolactinoma samples. Aberrant mRNAs that contain a premature termination codon are affected by NMD to prevent potentially harmful effects of their translation to proteins [34]. Interestingly, mutant SF3B1 induced the inclusion of 16 intronic nucleotides in exon 22 by using an upstream cryptic 3′ splice site in human sequences (Fig. 3A), and the nucleotide distances were not multiples of 3. This result indicated that the aberrant transcripts may generate a premature termination codon due to the shift of reading frame and therefore degraded by NMD [32]. Although mutant SF3B1 inducing different splicing events in rats and humans, *DLG1* mRNA and protein were downregulated in both models. Remarkably, pladienolide-B, a natural compound, is a spliceosome inhibitor and specifically targets the SF3B1 spliceosome subunit, and have been proven to inhibit the proliferation of many tumors [35, 36]. Particularly, Vázquez-Borrego MC et al. found that pladienolide-B markedly decreased cell proliferation of GH3 cells, which was similar to our results (data not shown) [37]. However, the effect and mechanism of pladienolide-B on *SF3B1*^*R625H*^ prolactinoma still need to be further explored.

Our data suggested that downregulating *DLG1* expression promote tumor cell migration and invasion. As previously reported, DLG1 was involved in mammalian tumorigenesis and was localized to adherens junctions in epithelial cells [38]. In mammals, *DLG1* appears to act as a tumor suppressor by affecting other polarity complexes, Myosin II activity, the actin cytoskeleton, and/or other signaling pathways [39–41]. Our studies indicated that knocking down *DLG1* induced EMT in tumor cells. EMT is well known to be related to the loss of cell polarity, specifically the loss of adherens junctions and enables the development of an invasive phenotype [42]. Consistent with our results, the study by Sugihara, T. et al. found that DLG1 loss was associated with poor prognosis in endometrial cancer and that knocking down *DLG1* accelerated tumor migration and invasion in vitro [29].

**Fig. 7:**
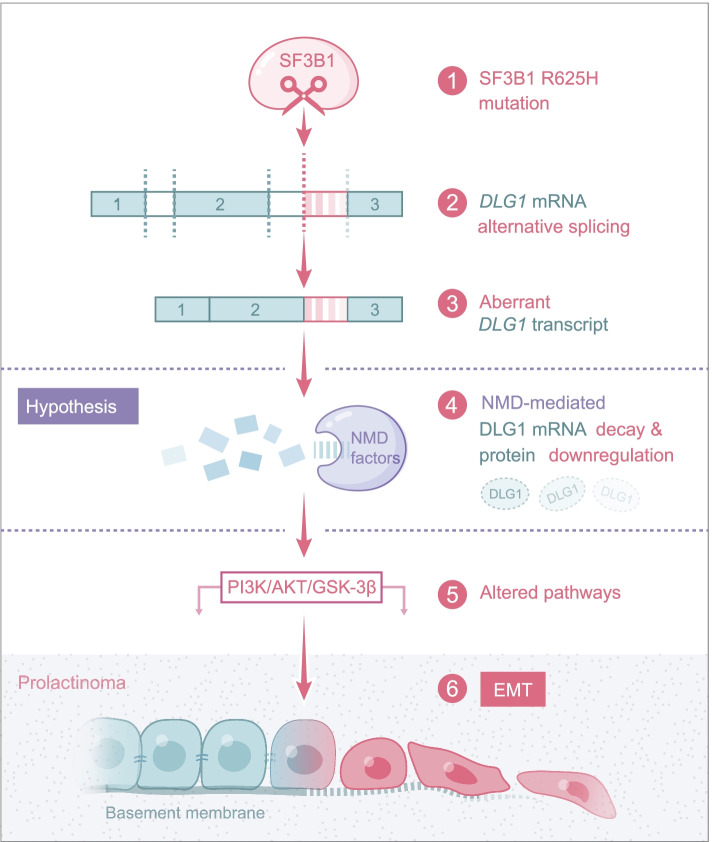
Schematic representation showing the proposed mechanisms through which the *SF3B1* mutation promotes the prolactinoma progression

Previous studies have illustrated that the AKT/GSK-3β/Snail axis is critical for the induction and maintenance of EMT [43, 44]. Here, we showed that mutant SF3B1 and decreased DLG1 expression activate the PI3K/AKT pathway, which may initiate migration and invasive via EMT (Fig. 7). It is known that p-AKT can suppress GSK-3β activity (through phosphorylation of Ser 9) and stabilize Snail [45]. Furthermore, DLG1 modulate the PI3K/Akt activity to precisely regulate regulatory T cell activity [46]. In the current study, we found that low DLG1 expression increased expression levels of p-AKT, p-GSK-3β, and Snail, while overexpression of DLG1 decreased these expression levels. Future work comprehensively defining the how DLG1 activates the PI3K/Akt signaling pathway in tumor cell may therefore be very important.

## Conclusions

In summary, we found the *SF3B1*-R625H mutation activates the PI3K/Akt pathway in prolactinoma by aberrant splicing of *DLG1*, promoting tumor invasion progression. Our findings provide the rationale for investigating new therapeutic strategies in patients with *SF3B1*-mutant prolactinoma.

## Supplementary Information

**Additional file 1: Table S1.** Primers used for qRT-PCR in this study. **Table S2.** The clinical data of 15 prolactinoma patients. **Table S3.** siRNA used in this study. **Table S4.** Antibodies used in this study. **Table S5.** Differentially expressed gene in *Sf3b1* mutant GH3 cells. **Table S6.** Differentially expressed gene in *SF3B1* mutant human prolactinoma. **Table S7.** Significant alternative splicing events in *Sf3b1* mutant GH3 cells. **Table S8.** Gene Ontology (GO) function of AS genes in *Sf3b1* mutant GH3 cells. **Table S9.** Significant alternative splicing events in *SF3B1* mutant human prolactinoma. **Table S10.** Gene Ontology (GO) function of AS genes in *SF3B1* mutant human prolactinoma. **Table S11.** Gene Ontology (GO) function of differentially expressed genes in Sf3b1 mutant GH3 cells. **Table S12.** KEGG pathway analysis of differentially expressed genes in Sf3b1 mutant GH3 cells. **Table S13.** KEGG pathway analysis of differentially expressed genes in SF3B1 mutant human prolactinoma.

**Additional file 2: Figure S1.** Generation of heterozygous *Sf3b1* R625H mutant GH3 cells. (A) Schematic diagram of the *Sf3b1* R625H CRISPR/Cas9-mediated strategy. (B) Visualization of the engineered mutation in the Integrative Genomic Viewer (IGV) browser: whole-exome sequencing reads overlapping the heterozygous mutation in the *Sf3b1* gene (c.1874G > A, c.1875A > T, p.R625H, frequency: 28.57%).

**Additional file 3: Figure S2.***SF3B1*-R625H alters the transcriptome in GH3 cells and human prolactinoma. (A) Volcano plot comparing mRNA expression between *Sf3b1* mutant vs. wild-type GH3 cells. (B) Heatmap of unsupervised clustering of differentially expressed genes in *Sf3b1* mutant vs. wild-type GH3 cells.. (C) Volcano plot comparing mRNA expression between *SF3B1* mutant vs. wild-type human prolactinoma.. (B) Heatmap of unsupervised clustering of differentially expressed genes in *SF3B1* mutant vs. wild-type human prolactinoma.

**Additional file 4: Figure S3.** Alternative splicing in *Sf3b1* mutant GH3 cells. (A-N) PCR products of the 13 genes (not including *Dlg1*) – *Pkd1l2*, *Myo15a*, *Tgif1*, *Rest*, *Mroh2a*, *Pipox*, *Emc1*, *Mapk15*, *Ccdc136*, *Tfb2m*, *Parp16*, *Parp4*, and *Cd44* were amplified from *Sf3b1*-mutant and WT GH3 cells. The cryptic and canonical transcripts were indicated by the short lines. *Gapdh* was used as a loading control.

**Additional file 5: Figure S4.** AS events in *SF3B1* mutant human prolactinoma and MRI scans of the estradiol-induced prolactinoma model. (A) Volcano plot showing the differential alternative splicing events in *SF3B1* mutant vs. wild-type human prolactinoma. Top 20 significant mis-spliced genes are indicated. (B) GO analysis of mis-spliced genes in *SF3B1* mutant vs. wild-type human prolactinoma; the top five ranked terms are shown. (C) MRI scan image before intra-pituitary injection (left) and after intra-pituitary injection (right).

**Additional file 6: Figure S5.** Mutant SF3B1 caused aberrant splicing of *DLG1* in 293 T and GH3 cells, and altered DLG1 expression in MMQ cells. (**A**) Schematic representation of the validated aberrant splicing of *DLG1* gene fragments by PCR. Schematic a represents the canonical DLG1 transcript and schematic b represents the aberrant DLG1 transcript. (B) The number of abnormal variant clones in the Ad-SF3B1-WT (2/48) and Ad-SF3B1-MT (7/43) groups, out of a total of 50 TA clones. (C) Sashimi plots of aberrant *Dlg1* splicing event in GH3 cells with or without *Sf3b1* mutations. (D) DLG1 expression level in MMQ cells infected with Ad-null, Ad-SF3B1^WT^, and Ad-SF3B1^R625H^.

## Data Availability

The datasets used and/or analyzed during the current study are available from the corresponding author on reasonable request.

## Abbreviations

MDS: Myelodysplastic syndrome
CLL: Chronic lymphocytic leukemia
NMD: Nonsense-mediated decay
FBS: Fetal bovine serum
FPKM: Fragments per kilobase per million mapped fragments
TPM: Transcripts per million
DEGs: Differentially expressed genes
GO: Gene Ontology
KEGG: Kyoto Encyclopedia of Genes and Genomes
BSA: Bovine serum albumin
MRI: Magnetic resonance imaging
MAGUKs: Membrane associated guanylate kinases
