# Supplementary figures and images for: The SF3B1R625H mutation promotes prolactinoma tumor progression through aberrant splicing of DLG1
Source: J Exp Clin Cancer Res. 2022 Jan 17;41:26. doi: 10.1186/s13046-022-02245-0 (PMC8762886; doi:10.1186/s13046-022-02245-0)

A

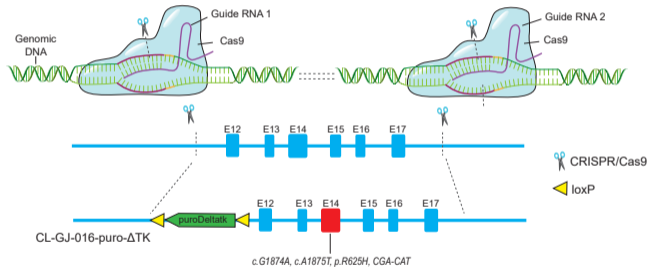

CRISPR/Cas9  
loxP

B

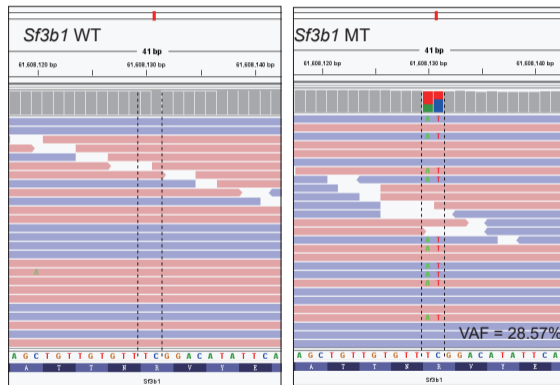

Supplement: Supplementary file 2 — Additional file 2: Figure S1. Generation of heterozygous Sf3b1 R625H mutant GH3 cells. (A) Schematic diagram of the Sf3b1 R625H CRISPR/Cas9-mediated strategy. (B) Visualization of the engineered mutation in the Integrative Genomic Viewer (IGV) browser: whole-exome sequencing reads overlapping the heterozygous mutation in the Sf3b1 gene (c.1874G > A, c.1875A > T, p.R625H, frequency: 28.57%). [file 13046_2022_2245_MOESM2_ESM.pdf]

A

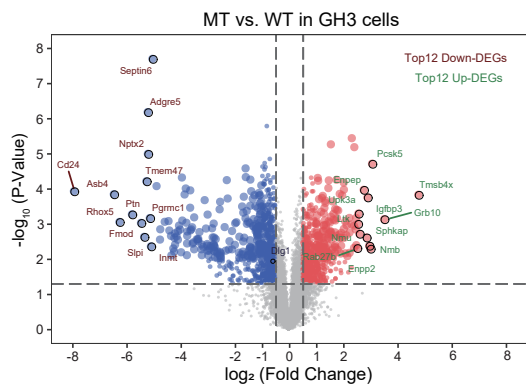

B

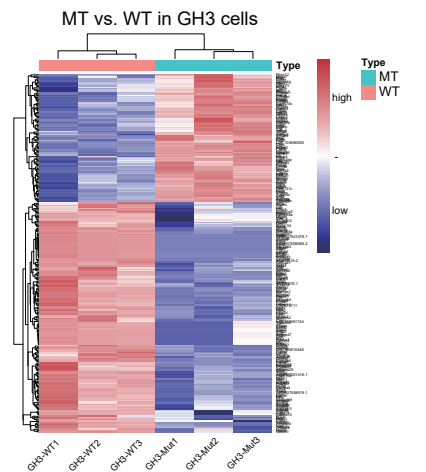

C

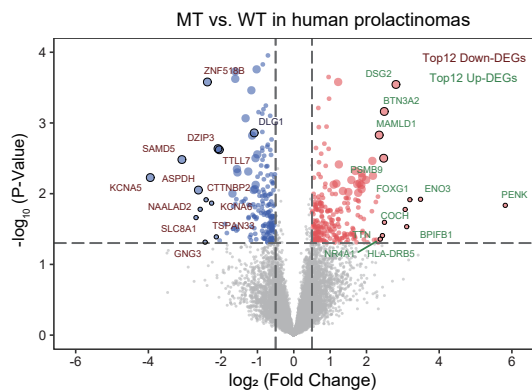

D

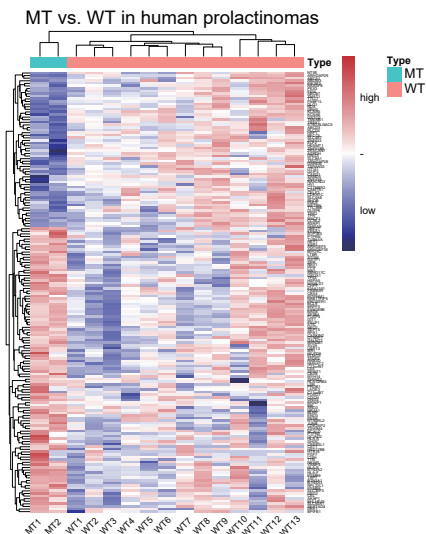

Supplement: Supplementary file 3 — Additional file 3: Figure S2.SF3B1-R625H alters the transcriptome in GH3 cells and human prolactinoma. (A) Volcano plot comparing mRNA expression between Sf3b1 mutant vs. wild-type GH3 cells. (B) Heatmap of unsupervised clustering of differentially expressed genes in Sf3b1 mutant vs. wild-type GH3 cells.. (C) Volcano plot comparing mRNA expression between SF3B1 mutant vs. wild-type human prolactinoma.. (B) Heatmap of unsupervised clustering of differentially expressed genes in SF3B1 mutant vs. wild-type human prolactinoma. [file 13046_2022_2245_MOESM3_ESM.pdf]

**A**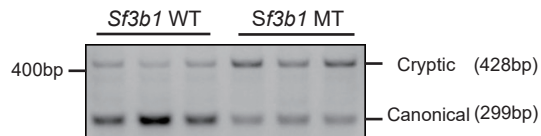**C**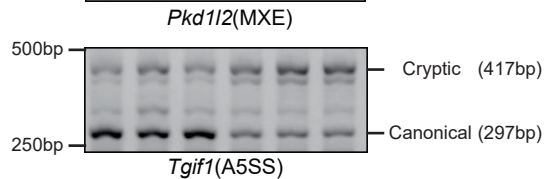**E**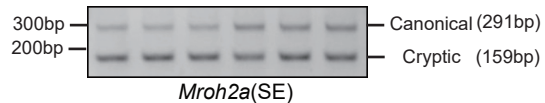**G**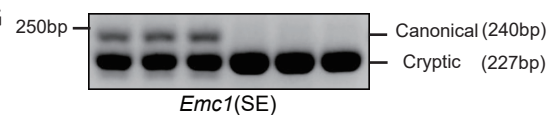**I**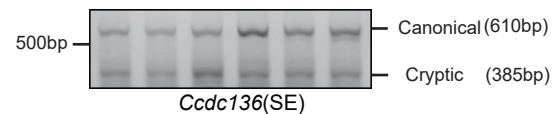**K**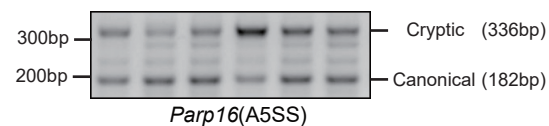**M**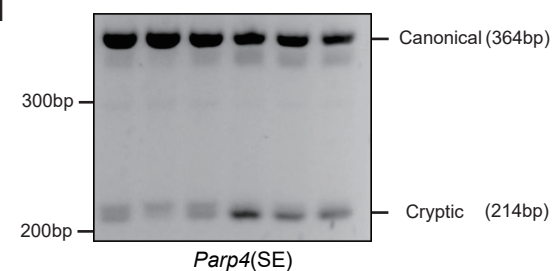**B**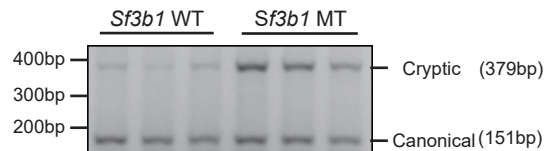**D**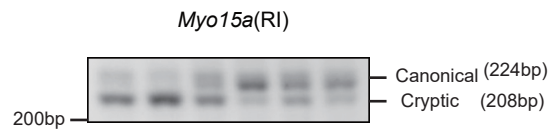**F**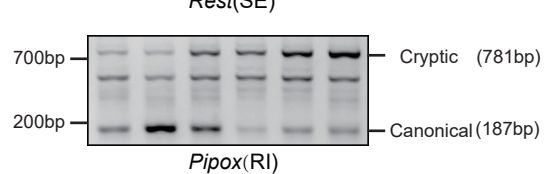**H**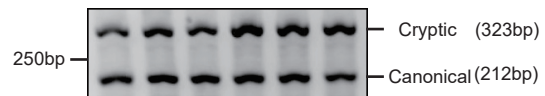**J**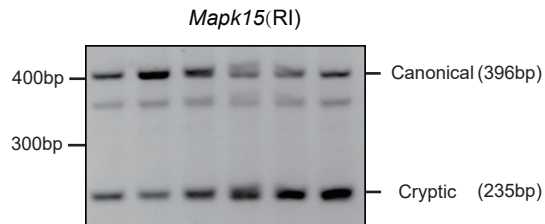**L**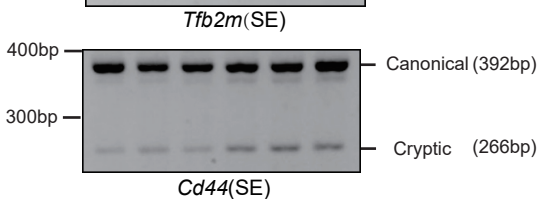**N**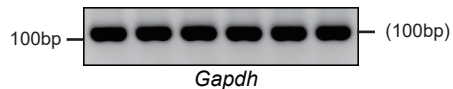

Supplement: Supplementary file 4 — Additional file 4: Figure S3. Alternative splicing in Sf3b1 mutant GH3 cells. (A-N) PCR products of the 13 genes (not including Dlg1) – Pkd1l2, Myo15a, Tgif1, Rest, Mroh2a, Pipox, Emc1, Mapk15, Ccdc136, Tfb2m, Parp16, Parp4, and Cd44 were amplified from Sf3b1-mutant and WT GH3 cells. The cryptic and canonical transcripts were indicated by the short lines. Gapdh was used as a loading control. [file 13046_2022_2245_MOESM4_ESM.pdf]

A

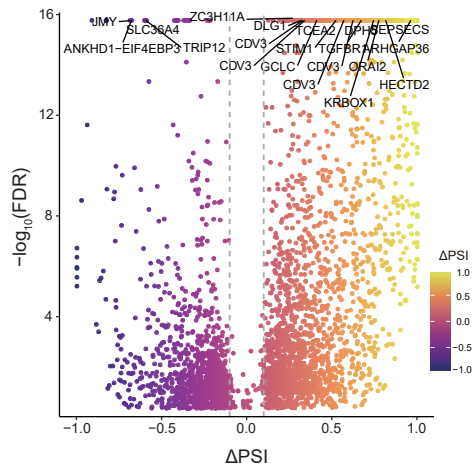

B

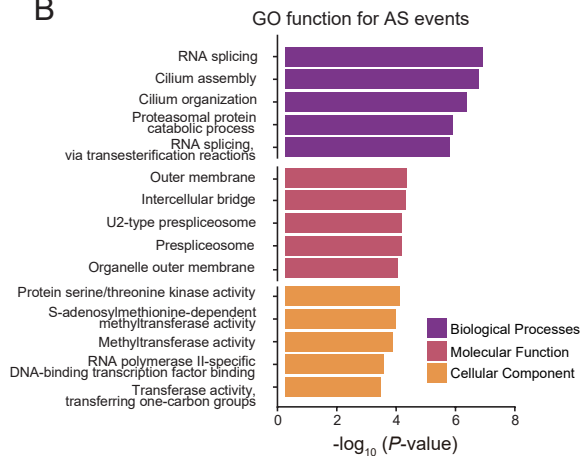

C

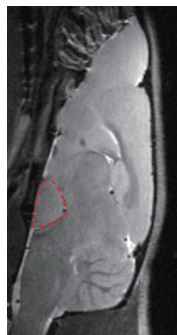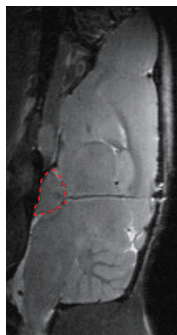

Needle  
track

Supplement: Supplementary file 5 — Additional file 5: Figure S4. AS events in SF3B1 mutant human prolactinoma and MRI scans of the estradiol-induced prolactinoma model. (A) Volcano plot showing the differential alternative splicing events in SF3B1 mutant vs. wild-type human prolactinoma. Top 20 significant mis-spliced genes are indicated. (B) GO analysis of mis-spliced genes in SF3B1 mutant vs. wild-type human prolactinoma; the top five ranked terms are shown. (C) MRI scan image before intra-pituitary injection (left) and after intra-pituitary injection (right). [file 13046_2022_2245_MOESM5_ESM.pdf]

A

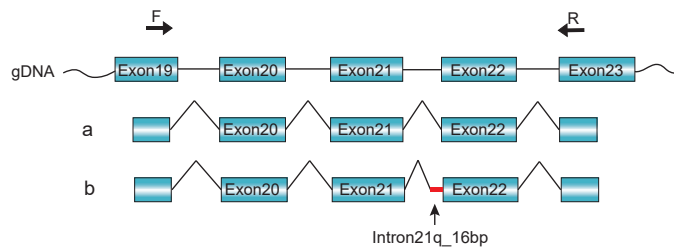

B

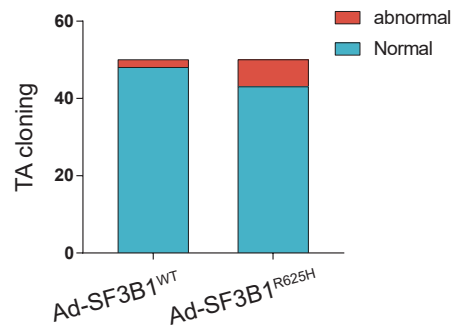

C

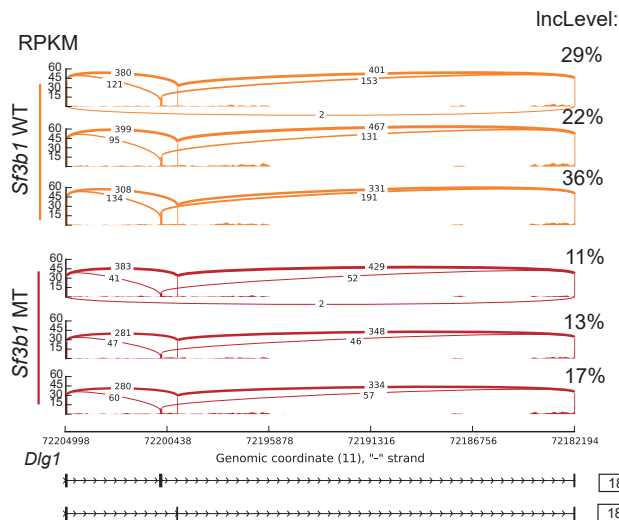

D

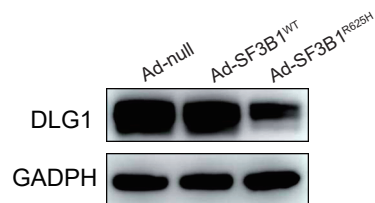

Supplement: Supplementary file 6 — Additional file 6: Figure S5. Mutant SF3B1 caused aberrant splicing of DLG1 in 293 T and GH3 cells, and altered DLG1 expression in MMQ cells. (A) Schematic representation of the validated aberrant splicing of DLG1 gene fragments by PCR. Schematic a represents the canonical DLG1 transcript and schematic b represents the aberrant DLG1 transcript. (B) The number of abnormal variant clones in the Ad-SF3B1-WT (2/48) and Ad-SF3B1-MT (7/43) groups, out of a total of 50 TA clones. (C) Sashimi plots of aberrant Dlg1 splicing event in GH3 cells with or without Sf3b1 mutations. (D) DLG1 expression level in MMQ cells infected with Ad-null, Ad-SF3B1WT, and Ad-SF3B1R625H. [file 13046_2022_2245_MOESM6_ESM.pdf]
